# Examining the complementarity between the ERIC compilation of implementation strategies and the behaviour change technique taxonomy: a qualitative analysis

**DOI:** 10.1186/s13012-022-01227-2

**Published:** 2022-08-19

**Authors:** Sheena McHugh, Justin Presseau, Courtney T. Luecking, Byron J. Powell

**Affiliations:** 1 School of Public Health, University College Cork, Western Gateway Building, Western Rd, Cork, Ireland; 2 School of Epidemiology & Public Health, University of Ottawa and Clinical Epidemiology Program, Ottawa Hospital Research Institute, Ottawa, Canada; 3 Department of Dietetics and Human Nutrition, College of Agriculture, Food and Environment, University of Kentucky, Lexington, USA; 4 Center for Mental Health Services Research, Brown School, Washington University in St. Louis, St. Louis, Missouri USA; 5 Center for Dissemination & Implementation, Institute for Public Health, Washington University in St. Louis, St. Louis, Missouri USA; 6 Division of Infectious Diseases, John T. Milliken Department of Medicine, Washington University School of Medicine, St. Louis, Missouri USA

**Keywords:** Implementation strategies, Behaviour change, Taxonomy, Intervention content, Implementation research reporting

## Abstract

**Background:**

Efforts to generate evidence for implementation strategies are frustrated by insufficient description. The Expert Recommendations for Implementing Change (ERIC) compilation names and defines implementation strategies; however, further work is needed to describe the actions involved. One potentially complementary taxonomy is the behaviour change techniques (BCT) taxonomy. We aimed to examine the extent and nature of the overlap between these taxonomies.

**Methods:**

Definitions and descriptions of 73 strategies in the ERIC compilation were analysed. First, each description was deductively coded using the BCT taxonomy. Second, a typology was developed to categorise the extent of overlap between ERIC strategies and BCTs. Third, three implementation scientists independently rated their level of agreement with the categorisation and BCT coding. Finally, discrepancies were settled through online consensus discussions. Additional patterns of complementarity between ERIC strategies and BCTs were labelled thematically. Descriptive statistics summarise the frequency of coded BCTs and the number of strategies mapped to each of the categories of the typology.

**Results:**

Across the 73 strategies, 41/93 BCTs (44%) were coded, with ‘restructuring the social environment’ as the most frequently coded (*n*=18 strategies, 25%). There was direct overlap between one strategy (*change physical structure and equipment*) and one BCT (‘restructuring physical environment’). Most strategy descriptions (*n*=64) had BCTs that were clearly indicated (*n*=18), and others where BCTs were probable but not explicitly described (*n*=31) or indicated multiple types of overlap (*n*=15). For some strategies, the presence of additional BCTs was dependent on the form of delivery. Some strategies served as examples of broad BCTs operationalised for implementation. For eight strategies, there were no BCTs indicated, or they did not appear to focus on changing behaviour. These strategies reflected preparatory stages and targeted collective cognition at the system level rather than behaviour change at the service delivery level.

**Conclusions:**

This study demonstrates how the ERIC compilation and BCT taxonomy can be integrated to specify active ingredients, providing an opportunity to better understand mechanisms of action. Our results highlight complementarity rather than redundancy. More efforts to integrate these or other taxonomies will aid strategy developers and build links between existing silos in implementation science.

**Supplementary Information:**

The online version contains supplementary material available at 10.1186/s13012-022-01227-2.

Contributions to the literature

- There are calls for greater integration and comparison of approaches in implementation science to avoid silos within the field.
- Examining the overlap between the ERIC compilation of implementation strategies and the behaviour change technique (BCT) taxonomy, we identified complementarity rather than redundancy.
- We specified most ERIC strategies in more detail using BCTs. Integrating the BCT taxonomy encourages consideration of actions, targets, and potential mechanisms of change.
- Some ERIC strategies provide contextual examples of how broadly defined BCTs could be operationalised for implementation.
- These taxonomies can be leveraged to enhance the reporting, replication, and synthesis of strategies.

## Background

Implementation strategies ‘have unparalleled importance in implementation science’ [1] and generating evidence for their effectiveness is a priority for the field [2, 3]. Implementation strategies are defined as ‘methods or techniques used to enhance the adoption, implementation, and sustainability of a clinical program or practice” [1] (Table 1). The opportunity to replicate strategies, tailored to local contexts where needed, and build cumulative knowledge, is partly limited by inconsistent labelling and insufficient description [4]. To address the problem, several taxonomies have been developed to (a) provide a standardised language for describing and reporting interventions and (b) provide a guide for those studying and those seeking to solve implementation problems [5–9]. These taxonomies can be used to describe the components of multifaceted and multilevel implementation interventions with varying degrees of detail [9]. However, there has been little exploration of the potential overlap, duplication, or complementarity between these taxonomies. Failure to consider this could exacerbate the problem of inconsistent labelling that these taxonomies were designed to address by contributing new terminology instead of synthesising and exploring connections between existing labels. It could also limit opportunities to synthesise results across studies that use different taxonomies.

**Table 1:** Definitions of key terms

**Taxonomy**	The scientific process of classifying things (arranging them into groups) [10].
**Implementation strategy**	Method or technique used to enhance the adoption, implementation, and sustainment of an evidence-based intervention [1].
**Behaviour change technique (BCT)**	Observable, replicable, and irreducible component of an intervention that has the potential to change behaviour. A technique is proposed to be an ‘active ingredient’ in an intervention. BCTs can be used alone or in combination and in a variety of formats [6].
**Implementation intervention**	The terminology to describe a collection of implementation activities is inconsistent. The collective noun has been named a ‘package’ of strategies [11] or an implementation intervention [12, 13]. We have selected the latter term to describe a collection of implementation strategies, based on the definition ‘an implementation intervention is defined as any type of strategy(s) that is designed to support a clinical intervention’ [14].

The Expert Recommendations of Implementation Change (ERIC) compilation was developed to provide a system to classify and organise the myriad of implementation strategies being used in research and practice [7, 15]. Building on a review of the health and mental health literatures, the initial ERIC compilation was developed using a modified Delphi process with a panel of experts from implementation science and clinical practice in North America, many of whom were affiliated with the Veterans Health Administration [15]. The project established consensus among researchers and practitioners for a common nomenclature for 73 implementation strategies [7]. In a subsequent study, concept mapping was used to organise the 73 implementation strategies into nine groups [16]. Since then, the ERIC compilation has been widely used in health implementation research and practice and has been adapted for other settings (e.g., schools [17, 18]), applied to particular types of interventions (e.g., digital mental health interventions [19]), and served as a foundation for expanding the range of specific implementation strategies (e.g., financing strategies [20]). However, several primary studies and reviews continue to note insufficient description of the content and format of popular strategies such as the use of local opinion leaders [21], continuing professional development [22], and development of education materials [23].

Most reporting guidelines recommend clear reporting of intervention content. The AIMD (Aims, Ingredients, Mechanism, Delivery) meta-framework validation project found that 95% of major reporting guidelines recommend the description of intervention ingredients defined as ‘the observable, replicable, and irreducible aspects of an intervention’ [24]. The Template for Intervention Description and Replication (TIDieR) checklist promotes the reporting of procedures and activities used in an intervention, including any activities to enable or support an intervention [25]. Similarly, the Standards for Reporting Implementation Studies (StaRI) Statement recommends dual reporting of the clinical, healthcare, or public health intervention being implemented and the implementation strategy [26]. Recommendations for specifying implementation strategies advise description of ‘the actions, steps, or processes and sequences of behaviour’ needed to enact a strategy using ‘dynamic verb statements’ that ideally should be behaviourally defined a priori [1].

One potentially complementary taxonomy, designed in part to enhance the intervention description, is the behaviour change technique (BCT) taxonomy (v1) [6]. The BCT taxonomy contains 93 discrete techniques. A BCT is defined as an ‘observable, replicable, and irreducible component of an intervention’ that has the potential to change behaviour [6], echoing the TIDieR definition of an intervention ingredient [27]. The BCT taxonomy was first developed in 2008, as a cross-behaviour classification system, using consensus methods and iterative reliability testing with international behaviour change experts [6]. It is now one of the most common classification systems used for describing activities in behaviour change interventions including interventions to support implementation and change professional practice in healthcare [13, 27–30]. The BCT taxonomy (v1) underpins the behaviour change wheel, a multistage framework for designing behaviour change interventions [31]. Within this approach, it is used to identify intervention content which can best serve intervention functions such as education and enablement.

Both the ERIC compilation and BCT taxonomy have been used to describe the ‘how to’ of implementation, albeit their applications differ in the scope of activities included and level of detail used to describe those activities. The ERIC compilation focusses primarily on team and organisational level strategies while the BCT Taxonomy focusses on change in individual or group behaviour, where the individual/groups may be at different and multiple organisational levels. Given that most implementation efforts require multilevel interventions, it is important to explore whether and how we can combine these approaches. We could identify few studies that have integrated or linked the ERIC compilation and BCT taxonomy to describe implementation in action [32, 33]. This is in contrast to the combined use of determinant frameworks to identify barriers and enablers in implementation science [34]. The proliferation of theories, models, and frameworks is a common criticism of implementation science; over time researchers become familiar with a particular approach and stick with it. To advance implementation science, there are calls for greater integration and comparison of approaches to avoid silos within the field [35].

The objective of our analysis was to examine the extent and nature of the overlap between the ERIC compilation [7] and the BCT taxonomy (v1) [6]. We chose to compare these two taxonomies as they are commonly used approaches to describe implementation interventions originating from different expert groups, they are typically applied separately, and they have varying levels of granularity in their descriptions suggesting scope for integration*.* By examining the potential links between these taxonomies, we can move from general descriptions of implementation strategies to more detailed and consistent descriptions of their content. By examining the overlap, we can move beyond differences in labels and bridge siloed approaches to designing and reporting implementation interventions.

## Methods

### Design

We conducted a qualitative content analysis of ERIC implementation strategy definitions to identify the inclusion of, or overlap with, BCTs. The study is reported in line with the COREQ reporting guidelines for qualitative studies (Additional file 1).

### Step 1: Content analysis of ERIC strategy descriptions

We used directed content analysis [36]. The BCT taxonomy (v1) was used as the pre-determined codebook used to code ERIC implementation strategy definitions [6]. It contains 93 techniques organised into 16 hierarchical categories. One author (SMcH) coded the definitions of the 73 ERIC strategies and the descriptions in the published in the ERIC paper and accompanying additional files [7]. The coder had completed online training in BCT coding and was experienced in using the BCT taxonomy to specify implementation interventions [29].

A sequential coding process was used, following the steps for coding intervention content outlined in the behaviour change wheel guide to designing interventions [37]. First, each ERIC strategy description was coded to identify one or more BCTs. Segments of text were checked against the BCT definition to assess whether that BCT was present or absent. The coder focussed on action words and verbs in the strategy definition to select the appropriate BCT. Where appropriate, multiple BCTs were assigned to the strategy description. On completion, all 93 BCT labels and definitions were re-considered for each of the strategy definitions. The coder noted her coding rationale throughout the process. Coding was managed using NVivo software (V12).

### Step 2: Developing a classification system

Using the results of the coding in step 1, a typology with five a priori categories was developed to categorise the nature of the overlap between ERIC strategies and BCTs. A typology is a formal system for classifying multifaceted complex phenomena according to a set of common conceptual dimensions in order to increase the clarity in defining and comparing complex phenomena [38]. The terms ‘typology’ and ‘taxonomy’ are often used interchangeable in the literature to describe this type of analytic output. We use the term ‘typology’ to avoid confusion with the BCT taxonomy used in the analysis. Also, it has been suggested that typologies are conceptually developed as is the case here, while taxonomies are empirically derived configurations [39].

The five ‘types’ or categories in the typology were developed initially by examining the patterns of overlap or links between ERIC strategies and BCTs (by SMcH). Each category was given a label and coding definition. Categories were reviewed by all authors and further refinements were made to the category labels and definitions. The typology consisted of five categories of overlap (Table 2). First, there were instances of *direct 1-1 overlap* between an ERIC strategy and a BCT, allowing for some differences in terminology. Second, there were instances where at least one *clear BCT was indicated in the ERIC strategy description* which could be used to guide initial operationalisation. Third, there were instances where at least *one probable BCT(s) was indicated in the ERIC strategy description*, that is to say the BCT was logically indicated but was not clearly or explicitly stated. Fourth, there were instances where *no BCTs were clearly indicated* in the ERIC strategy definition or description. Fifth, some ERIC strategies *did not appear to target behaviour change* to support implementation; thus, an underlying behavioural target was not clear.

**Table 2:** Data-driven a priori typology developed to classify the nature and extent of overlap between ERIC strategies and BCTs

Type of overlap	Definition
Direct 1-1 overlap between ERIC implementation strategy and BCT	The ERIC strategy equates to a BCT, allowing for differences in terminology.
Clear BCT indicated in ERIC implementation strategy description	There is a BCT clearly indicated in the ERIC strategy and could be used to guide initial operationalisation. Other BCTs are possible as part of the strategy but not clearly indicated.
Probable BCT indicated in ERIC implementation strategy description	This BCT is logically indicated in the ERIC strategy given its title, definition, and/or description but not clearly or explicitly. Other BCTs may be possible depending on how the strategy is operationalised.
No BCTs indicated in ERIC implementation strategy description	There are no BCTs indicated directly or logically in the strategy definition or description, despite its focus on implementation.
ERIC implementation strategy not targeting behaviour change	The ERIC strategy does not appear to focus on behaviour change to support implementation.

It is important to note that the typology was applied to describe the pattern of overlap between a BCT and aspects of an ERIC strategy description. During analysis, it became apparent that more than one type of overlap could apply within a single strategy description, depending on the BCT being considered. In these cases, a strategy was categorised as having *multiple types of overlap indicated.* However, as this was not one of the five a priori categories in the typology, we present this in the results section.

### Step 3: Independent rating

The first round of coding (by SMcH) was tabulated in Excel. Three implementation scientists (JP, BP, CL) independently rated their level of agreement with the BCT(s) coded to an ERIC strategy description and the type of overlap assigned. They rated their agreement on a scale of 0 (complete disagreement) to 10 (complete agreement). They also provided suggestions for BCTs to be removed or added, and changes to categorisations. All coders were had experience coding qualitative data and expertise in both frameworks. Average agreement scores were calculated for each BCT and feedback was collated (by SMH). Coded BCTs with agreement scores of ≥7 were deemed to have reached consensus. A revised list of ERIC strategies was compiled, comprising those that had agreement score of <7 or where suggestions were made to reclassify a strategy or add/remove additional BCTs.

### Step 4: Review

The remaining undecided ERIC strategies were reviewed by team. Two virtual meetings were held to review discrepancies in agreement and suggested changes to classification and BCT coding. In cases where team members disagreed, we revisited the full ERIC description and examples given in the ERIC compilation and considered its grouping and function as a change strategy (e.g., is the function to educate, to enable, or incentivise practitioners). During team discussions, groups of ERIC strategies were reviewed together to ensure consistency of coding. When reviewing groups of strategies, we identified additional patterns of complementarity between ERIC strategies and BCTs. These patterns were labelled thematically. The results were drafted and circulated to all co-authors and final revisions were made to the classification and BCTs coded.

### Data analysis

Across ERIC strategies, we quantified the frequency of BCT occurring (e.g., X BCT occurred 10 times across strategies). We estimated the number of BCT groupings represented in ERIC strategies (*n*=16 possible hierarchical groupings in the BCT taxonomy). We quantified the total number of ERIC strategies mapped to each of the five types of overlap in the typology (Table 2). The results were compared across the nine groups of strategies in the ERIC compilation: (1) use evaluative and iterative strategies, (2) provide interactive assistance, (3) adapt and tailor to context, (4) develop stakeholder relationships, (5) train and educate stakeholders, (6) support clinicians, (7) engage consumers, (8) utilise financial strategies, and (9) change infrastructure [16].

## Results

The results are organized into three sections. First, we summarize the number of BCTs coded to ERIC strategies. Second, we summarize the number of ERIC strategies assigned to the types of overlap. Finally, we describe additional patterns of complementarity between ERIC strategies and BCTs developed during the analysis and provide examples to illustrate those themes. ERIC implementation strategies titles are written in italics (e.g., *remind clinicians*), and BCTs are reported using quotation marks (e.g., ‘prompts and cues’).

### Characteristics of coded BCTs

**Fig. 1:**
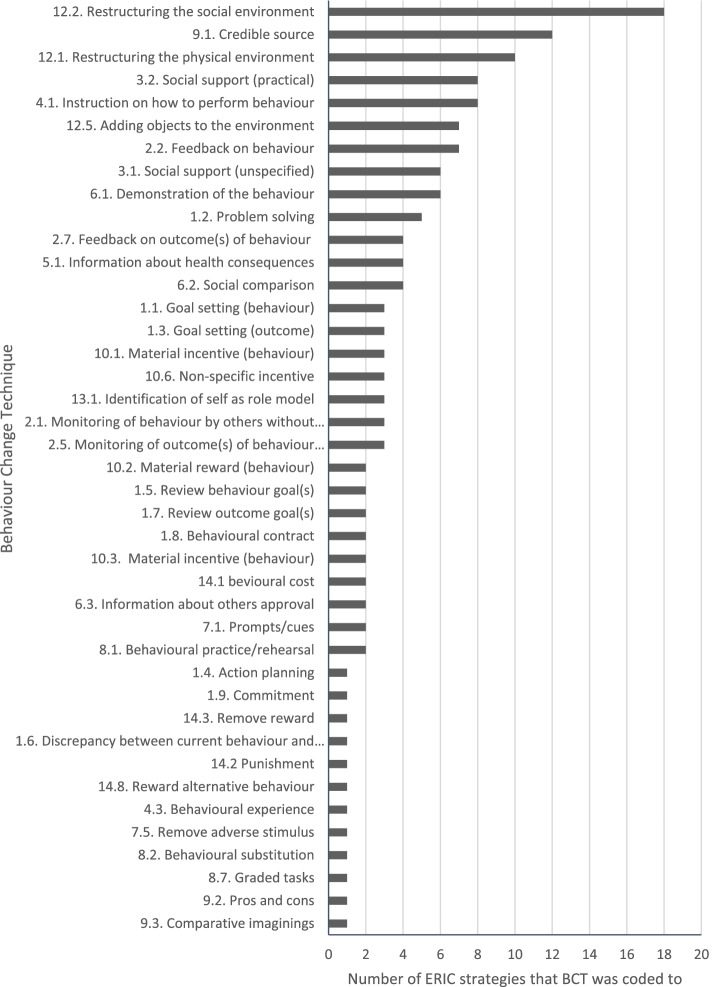
Frequency of BCTs coded in ERIC implementation strategy descriptions

Of the 73 ERIC strategies analysed, BCTs were coded 150 times. Overall, 41 out of the 93 BCTs (44%) were identified. At least one BCT was coded from 13 of the 16 possible groupings from the BCT Taxonomy; no BCTs were coded from regulation, self-belief, or covert learning groups. The most frequently coded BCT was ‘restructuring the social environment’ (*n*=18 strategies, coded in 25% of strategies) (Fig. 1).

### Types of overlap between strategies and BCTs

Table 3 outlines the number of strategies categorised to each type of overlap in the typology. One strategy was categorized as directly overlapping with a BCT; the strategy to *change physical structure and equipment* equates to ‘restructuring the physical environment’. Most ERIC strategy descriptions (*n*=64) contained BCTs that were clearly indicated or BCTs that were probable or indicated multiple types of overlap. This pattern was reflected across the nine ERIC strategy groups. Four strategies were categorised as having no BCTs indicated in the strategy definition or detailed description*.* Four strategies were categorised as not focusing on behaviour change to support implementation.

**Table 3:** Number of ERIC strategies categorised to each type of overlap, per group, and overall (*n*=73)

Type of overlap	ERIC grouping									Total strategies per category
	Group 1: Use evaluative and iterative strategies (*n*=10)	Group 2: Provide interactive assistance (*n*=4)	Group 3: Adapt & tailor to context (*n*=4)	Group 4: Develop stakeholder relationships (*n*=17)	Group 5: Train & educate stakeholders (*n*=11)	Group 6: Support clinicians (*n*=5)	Group 7: Engage consumers (*n*=5)	Group 8: Utilise financial strategies (*n*=9)	Group 9: Change infrastructure (*n*=8)	
Direct 1-1^a^	0	0	0	0	0	0	0	0	1	1
Clear BCT indicated	2	0	0	6	2	4	0	3	1	18
Probable BCT indicated	4	1	3	5	4	0	5	5	4	31
Multiple types of overlap indicated	2	3	0	5	2	1	0	1	1	15
No BCTs indicated	1	0	1	0	2	0	0	0	0	4
ERIC strategy not targeting behaviour change	1	0	0	1	1	0	0	0	1	4

^a^This strategy also included a BCT that was clearly indicated in the description. It is assigned here to the direct 1-1 overlap category rather than ‘multiple types of overlap’ category

#### Direct 1-1 overlap between ERIC  strategy and BCT

There was one instance where the ERIC implementation strategy directly overlapped with a BCT. The strategy to *change physical structure and equipment* directly overlapped with ‘restructuring the physical environment’. The strategy and BCT referred to the same change at the same level, albeit using different terminology.

#### Clear and probable BCTs indicated in ERIC strategy descriptions

Most ERIC strategy descriptions (*n*=64) contained BCTs that were clearly indicated, BCTs that were probable, or indicated multiple types of overlap (Table 4).

**Table 4:** ERIC strategies with BCTs coded (where appropriate) and type of overlap indicated

ERIC group & strategy	Strategy definition	No. of BCTs	BCT identified *[grouping]*	Overlap between ERIC strategy & BCT
**Group 1: Use evaluative and iterative strategies**			*1.Goals and planning* *2. Feedback and monitoring* *4.Shaping knowledge* *6.Comparison of behaviour* *8.Repetition and substitution* *9.Comparison of outcomes* *12.Antecedents*	
Assess for readiness and identify barriers and facilitators	Assess various aspects of an organization to determine its degree of readiness to implement, barriers that may impede implementation, and strengths that can be used in the implementation effort	0	-	No BCTs indicated in ERIC strategy description
Audit and provide feedback	Collect and summarize clinical performance data over a specified time period and *give it* to clinicians and administrators to monitor, evaluate, and modify provider behaviour	2	2.2. Feedback on behaviour	Clear BCT indicated in ERIC strategy
			2.7. Feedback on outcome(s) of behaviour	Clear BCT indicated in ERIC strategy
Develop a formal implementation blueprint	Develop a formal implementation blueprint that includes all goals and strategies. The blueprint should include the following: 1) aim/purpose of the implementation; 2) *scope of the change* (e.g., what organizational units are affected); 3) *timeframe and milestones*; and 4) appropriate performance/progress measures. Use and update this plan to guide the implementation effort over time.	3	1.4. Action planning	Clear BCT indicated in ERIC strategy
			1.1. Goal setting (behaviour)	Clear BCT indicated in ERIC strategy
			1.3. Goal setting (outcomes)	Clear BCT indicated in ERIC strategy
Conduct cyclical small tests of change	Implement changes in a cyclical fashion using small tests of change before taking changes system-wide. Tests of change benefit from systematic measurement, and results of the tests of change are studied for insights on how to do better. This process continues serially over time, and refinement is added with each cycle	7	2.2. Feedback on behaviour	Probable BCT indicated in ERIC strategy
			2.7 Feedback on outcomes of behaviour	Probable BCT indicated in ERIC strategy
			1.5. Review behaviour goal(s)	Probable BCT indicated in ERIC strategy
			1.7. Review outcome goal(s)	Probable BCT indicated in ERIC strategy
			1.1. Goal setting (behaviour)	Probable BCT indicated in ERIC strategy
			1.3. Goal setting (outcome)	Probable BCT indicated in ERIC strategy
			4.3. Behavioural experience	Clear BCT indicated in ERIC strategy
Develop and implement tools for quality monitoring	Develop, test, and introduce into quality-monitoring systems the right input—the appropriate language, protocols, algorithms, standards, and measures (of processes, patient/consumer outcomes, and implementation outcomes) that are often specific to the innovation being implemented	2	12.1. Restructuring the physical environment	Probable BCT indicated in ERIC strategy
			12.5. Adding objects to the environment	Probable BCT indicated in ERIC strategy
Develop and organize quality monitoring systems	Develop and organize systems and procedures that monitor clinical processes and/or outcomes for the purpose of quality assurance and improvement	5	12.1. Restructuring the physical environment	Probable BCT subsumed under ERIC strategy
			2.1. Monitoring of behaviour by others without feedback	Clear BCT subsumed under ERIC strategy
			2.5. Monitoring of outcome(s) of behaviour without feedback	Clear BCT subsumed under ERIC strategy
			2.2. Feedback on behaviour	Probable BCT subsumed under ERIC strategy
			2.7. Feedback on outcome(s) of behaviour	Probable BCT subsumed under ERIC strategy
Obtain and use patients/consumers and family feedback	Develop strategies to increase patient/consumer and family feedback on the implementation effort	3	2.2. Feedback on behaviour	Probable BCT indicated in ERIC strategy
			9.1. Credible source	Probable BCT indicated in ERIC strategy
			6.3. Information about others approval	Probable BCT indicated in ERIC strategy
Purposefully re-examine the implementation	Monitor progress and adjust clinical practices and implementation strategies to continuously improve the quality of care	5	2.1. Monitoring of behaviour by others without feedback	Probable BCT indicated in ERIC strategy
			2.5. Monitoring of outcome(s) of behaviour without feedback	Probable BCT indicated in ERIC strategy
			1.5. Review behaviour goal(s)	Probable BCT indicated in ERIC strategy
			1.7. Review outcome goal(s)	Probable BCT indicated in ERIC strategy
			1.6. Discrepancy between current behaviour and goal	Probable BCT indicated in ERIC strategy
Stage implementation scale up	Phase implementation efforts by starting with small pilots or demonstration projects and gradually move to a system wide rollout	1	8.7. Graded tasks	Probable BCT indicated in ERIC strategy
Conduct local need assessment	Collect and analyze data related to the need for the innovation	0	-	ERIC strategy not targeting behaviour change
**Group 2: Provide interactive assistance**			*1.Goals and planning* *3. Social support* *4.Shaping knowledge* *9.Comparison of outcomes* *12.Antecedents*	
Centralize technical assistance	Develop and use a centralized system to deliver technical assistance focused on implementation issues	2	12.1. Restructuring the physical environment	Probable BCT indicated in ERIC strategy
			3.2. Social support (practical)	Probable BCT indicated in ERIC strategy
Provide local technical assistance	Develop and use a system to deliver technical assistance focused on implementation issues using local personnel	2	12.2. Restructuring the social environment	Clear BCT indicated in ERIC strategy
			3.2. Social support (practical)	Probable BCT indicated in ERIC strategy
Facilitation	A process of interactive problem solving and support that occurs in a context of a recognized need for improvement and a supportive interpersonal relationship	3	3.1. Social support (unspecified)	Clear BCT indicated in ERIC strategy
			1.2. Problem solving	Clear BCT indicated in ERIC strategy
			3.2. Social support (practical)	Probable BCT indicated in ERIC strategy
Provide clinical supervision	Provide clinicians with ongoing supervision focusing on the innovation.	3	12.2. Restructuring the social environment	Clear BCT indicated in ERIC strategy
			9.1. Credible source	Probable BCT indicated in ERIC strategy
			4.1 Instruction on how to perform behaviour	Probable BCT indicated in ERIC strategy
**Group 3: Adapt and tailor to context**			*1.Goals and planning* *3.Social Support* *9.Comparison of outcomes* *12.Antecedents*	
Use data experts	Involve, hire, and/or consult experts to inform management on the use of data generated by implementation efforts	2	9.1. Credible source	Probable BCT indicated in ERIC strategy
			3.2. Social support (practical)	Probable BCT indicated in ERIC strategy
Use data warehousing techniques	Integrate clinical records across facilities and organizations to facilitate implementation across systems	1	12.1. Restructuring the physical environment	Probable BCT indicated in ERIC strategy
Tailor strategies	Tailor the implementation strategies to address barriers and leverage facilitators that were identified through earlier data collection	1	1.2. Problem solving	Probable BCT indicated in ERIC strategy
Promote adaptability	Identify the ways a clinical innovation can be tailored to meet local needs and clarify which elements of the innovation must be maintained to preserve fidelity	0	-	No BCTs indicated in ERIC strategy description
**Group 4: Develop stakeholder interrelationships**			*1.Goals and planning* *2. Feedback and monitoring* *3.Social Support* *6.Comparison of behaviour* *8.Repetition and substitution* *9.Comparison of outcomes* *12.Antecedents* *13. Identity*	
Conduct local consensus discussions	Include local providers and other stakeholders in discussions that address whether the chosen problem is important and whether the clinical innovation to address it is appropriate	1	9.2. Pros and cons	Probable BCT indicated in ERIC strategy
Develop academic partnerships	Partner with a university or academic unit for the purposes of shared training and bringing research skills to an implementation project	1	12.2. Restructuring the social environment	Probable BCT indicated in ERIC strategy
Identify and prepare champions	Identify and prepare individuals who dedicate themselves to supporting, marketing, and driving through an implementation, overcoming indifference or resistance that the intervention may provoke in an organization	4	12.2. Restructuring the social environment	Clear BCT indicated in ERIC strategy
			9.1. Credible source	Clear BCT indicated in ERIC strategy
			3.1. Social support (unspecified)	Probable BCT indicated in ERIC strategy
			13.1. Identification of self as role model	Probable BCT indicated in ERIC strategy
Identify early adopters	Identify early adopters at the local site to learn from their experiences with the practice innovation	1	6.2. Social comparison	Probable BCT indicated in ERIC strategy
Inform local opinion leaders	Inform providers identified by colleagues as opinion leaders or “educationally influential” about the clinical innovation in the hopes that they will influence colleagues to adopt it	2	9.1. Credible source	Probable BCT indicated in ERIC strategy
			13.1 Identification of self as role model	Clear BCT indicated in ERIC strategy
Recruit, designate, and train for leadership	Recruit, designate, and train leaders for the change effort	1	12.2. Restructuring the social environment	Probable BCT indicated in ERIC strategy
Use an implementation advisor	Seek guidance from experts in implementation	2	9.1. Credible source	Probable BCT indicated in ERIC strategy
			3.1. Social support (unspecified)	Probable BCT indicated in ERIC strategy
Visit other sites	Visit sites where a similar implementation effort has been considered successful	4	6.2. Social comparison	Clear BCT indicated in ERIC strategy
			6.1. Demonstration of behaviour	Clear BCT indicated in ERIC strategy
			9.1. Credible source	Probable BCT indicated in ERIC strategy
			3.2. Social support (practical)	Clear BCT indicated in ERIC strategy
Model and simulate change	Model or simulate the change that will be implemented prior to implementation	2	8.1. Behavioural rehearsal	Probable BCT indicated in ERIC strategy
			9.3. Comparative imaginings	Clear BCT indicated in ERIC strategy
Promote network weaving	Identify and build on existing high-quality working relationships and networks within and outside the organization, organizational units, teams, etc. to promote information sharing, collaborative problem-solving, and a shared vision/goal related to implementing the innovation	3	12.2. Restructuring the social environment	Clear BCT indicated in ERIC strategy
			1.2. Problem solving	Probable BCT indicated in ERIC strategy
			1.3. Goal setting (outcome)	Probable BCT indicated in ERIC strategy
Organize clinician implementation team meetings	Develop and support teams of clinicians who are implementing the innovation and give them protected time to reflect on the implementation effort, share lessons learned, and support one another’s learning	2	12.2. Restructuring the social environment	Clear BCT indicated in ERIC strategy
			3.1. Social support (unspecified)	Clear BCT indicated in ERIC strategy
Build a coalition	Recruit and cultivate relationships with partners in the implementation effort	1	12.2. Restructuring the social environment	Clear BCT indicated in ERIC strategy
Obtain formal commitments	Obtain written commitments from key partners that *state what they will do* to implement the innovation	3	1.9. Commitment	Clear BCT indicated in ERIC strategy
			1.1. Goal setting (behaviour)	Clear BCT indicated in ERIC strategy
			1.8. Behavioural contract	Clear BCT indicated in ERIC strategy
Capture and share local knowledge	Capture local knowledge from implementation sites on how implementers and clinicians made something work in their setting and then share it with other sites	2	3.2. Social support (practical)	Clear BCT indicated in ERIC strategy
			6.2. Social comparison	Clear BCT indicated in ERIC strategy
Involve executive boards	Involve existing governing structures (e.g., boards of directors, medical staff boards of governance) in the implementation effort, including the review of data on implementation processes	3	12.2. Restructuring the social environment	Clear BCT indicated in ERIC strategy
			2.1. Monitoring of behaviour by others without feedback	Clear BCT indicated in ERIC strategy
			2.5. Monitoring outcome(s) of behaviour by others without feedback	Clear BCT indicated in ERIC strategy
Use advisory boards and workgroups	Create and engage a formal group of multiple kinds of stakeholders to provide input and advice on implementation efforts and to elicit recommendations for improvements	2	12.2. Restructuring the social environment	Clear BCT indicated in ERIC strategy
			3.2. Social support (practical)	Clear BCT indicated in ERIC strategy
Develop an implementation glossary	Develop and distribute a list of terms describing the innovation, implementation, and stakeholders in the organizational change	0		ERIC strategy not targeting behaviour change
**Group 5: Train and educate stakeholders**			*1.Goals and planning* *2. Feedback and monitoring* *3.Social support* *4.Shaping knowledge* *5.Natural consequences* *6.Comparison of behaviour* *8.Repetition and substitution* *9.Comparison of outcomes* *12.Antecedents* *13.Identity*	
Conduct educational meetings	Hold meetings targeted toward different stakeholder groups (e.g., providers, administrators, other organizational stakeholders, and community, patient/consumer, and family stakeholders) to teach them about the clinical innovation	3	4.1. Instruction on how to perform behaviour	Probable BCT indicated in ERIC strategy
			5.1. Information about health consequences	Probable BCT indicated in ERIC strategy
			6.1. Demonstration of the behaviour	Probable BCT indicated in ERIC strategy
Conduct educational outreach visits	Have a trained person meet with providers in their practice settings to educate providers about the clinical innovation with the intent of changing the provider’s practice	7	9.1. Credible source	Clear BCT indicated in ERIC strategy
			4.1. Instruction on how to perform behaviour	Probable BCT indicated in ERIC strategy
			5.1. Info about health consequences	Probable BCT indicated in ERIC strategy
			6.1. Demonstration of the behaviour	Probable BCT indicated in ERIC strategy
			2.2. Feedback on behaviour	Probable BCT indicated in ERIC strategy
			1.2. Problem solving	Probable BCT indicated in ERIC strategy
			8.2. Behavioural substitution	Probable BCT indicated in ERIC strategy
Create a learning collaborative	Facilitate the formation of groups of providers or provider organizations and foster a collaborative learning environment to improve implementation of the clinical innovation	2	12.2. Restructuring the social environment	Clear BCT indicated in ERIC strategy
			3.1. Social support (unspecified)	Probable BCT indicated in ERIC strategy
Conduct ongoing training	Plan for and conduct training in the clinical innovation in an ongoing way	3	4.1. Instruction on how to perform behaviour	Probable BCT indicated in ERIC strategy
			6.1. Demonstration of behaviour	Probable BCT indicated in ERIC strategy
			8.1. Behavioural practice/rehearsal	Probable BCT indicated in ERIC strategy
Develop educational materials	Develop and format manuals, toolkits, and other supporting materials in ways that make it easier for stakeholders to learn about the innovation and for clinicians to learn how to deliver the clinical innovation	0		No BCTs indicated in ERIC strategy description
Distribute educational materials	Distribute educational materials (including guidelines, manuals, and toolkits) in person, by mail, and/or electronically	2	4.1. Instruction on how to perform behaviour	Probable BCT indicated in ERIC strategy
			5.1. Information about health consequences	Probable BCT indicated in ERIC strategy
Use train-the-trainer strategies	Train designated clinicians or organizations to train others in the clinical innovation	4	6.1. Demonstration of the behaviour	Probable BCT indicated in ERIC strategy
			4.1. Instruction on how to perform behaviour	Probable BCT indicated in ERIC strategy
			9.1. Credible source	Probable BCT indicated in ERIC strategy
			13.1 Identification of self as role model	Probable BCT indicated in ERIC strategy
Provide ongoing consultation	Provide ongoing consultation with one or more experts in the strategies used to support implementing the innovation	4	3.1. Social support (unspecified)	Clear BCT indicated in ERIC strategy
			9.1. Credible source	Clear BCT indicated in ERIC strategy
			2.2. Feedback on behaviour	Clear BCT indicated in ERIC strategy
			4.1. Instruction on how to perform behaviour	Clear BCT indicated in ERIC strategy
Shadow other experts	Provide ways for key individuals to directly observe experienced people engage with or use the targeted practice change/innovation	1	6.1. Demonstration of behaviour	Clear BCT indicated in ERIC strategy
Make training dynamic	Vary the information delivery methods to cater to different learning styles and work contexts, and shape the training in the innovation to be interactive	0	-	No BCTs indicated in ERIC strategy description
Work with educational institutions	Encourage educational institutions to train clinicians in the innovation	0	-	Strategy does not focus on behaviour change for implementation
**Group 6: Support clinicians**			*2. Feedback and monitoring* *3.Social support* *7.Associations* *12.Antecedents*	
Facilitate relay of clinical data to providers	Provide as close to real-time data as possible about key measures of process/outcomes using integrated modes/channels of communication in a way that promotes use of the targeted innovation	4	2.2. Feedback on behaviour	Clear BCT indicated in ERIC strategy
			2.7. Feedback on outcome(s) of behaviour	Clear BCT indicated in ERIC strategy
			12.1. Restructuring the physical environment	Probable BCT indicated in ERIC strategy
			12.2. Restructuring the social environment	Probable BCT indicated in ERIC strategy
Create new clinical teams	Change who serves on the clinical team, adding different disciplines and different skills to make it more likely that the clinical innovation is delivered (or is more successfully delivered)	1	12.2. Restructuring the social environment	Clear BCT indicated in ERIC strategy
Develop resource sharing agreement	Develop partnerships with organizations that have resources needed to implement the innovation	2	12.2. Restructuring the social environment	Clear BCT indicated in ERIC strategy
			3.2. Social support (practical)	Clear BCT indicated in ERIC strategy
Remind clinicians	Develop reminder systems designed to help clinicians to recall information and/or prompt them to use the clinical innovation	2	7.1. Prompts/cues	Clear BCT subsumed under ERIC strategy
			12.5. Adding objects to the environment	Clear BCT subsumed under ERIC strategy
Revise professional roles	Shift and revise roles among professionals who provide care, and redesign job characteristics	1	12.2. Restructuring the social environment	Clear BCT indicated in ERIC strategy
**Group 7: Engage consumers**			*1.Goals and planning* *5.Natural consequences* *6.Comparison of behaviour* *7.Associations* *9.Comparison of outcomes* *12.Antecedents*	
Increase demand	Attempt to influence the market for the clinical innovation to increase competition intensity and to increase the maturity of the market for the clinical innovation	2	6.2. Social comparison	Probable BCT indicated in ERIC strategy
			9.1. Credible source	Probable BCT indicated in ERIC strategy
Involve patients/consumers and family members	Engage or include patients/consumers and families in the implementation effort	1	12.2. Restructuring the social environment	Probable BCT indicated in ERIC strategy
Intervene with patients/consumers to enhance uptake and adherence	Develop strategies with patients to encourage and problem solve around adherence	1	1.2. Problem solving	Probable BCT indicated in ERIC strategy
Prepare patients/consumers to be active participants	Prepare patients/consumers to be active in their care, to ask questions, and specifically to inquire about care guidelines, the evidence behind clinical decisions, or about available evidence-supported treatments	1	5.1. Information about health consequences	Probable BCT indicated in ERIC strategy
Use mass media	Use media to reach large numbers of people to spread the word about the clinical innovation	2	12.5. Adding objects to the environment	Probable BCT indicated in ERIC strategy
			7.1. Prompts/cues	Probable BCT indicated in ERIC strategy
**Group 8: Utilize financial incentives**			*1.Goals and planning* *10.Reward and threat* *12.Antecedents* *14.Scheduled consequences*	
Alter incentive/allowance structures	Work to incentivize the adoption and implementation of the clinical innovation	2	10.1. Material incentive (behaviour)	Clear BCT indicated in ERIC strategy
			10.6. Non-specific incentive	Probable BCT indicated in ERIC strategy
Place innovation on fee for service lists/formularies	Work to place clinical innovation on lists of actions for which providers can be reimbursed	1	10.2. Material reward (behaviour)	Clear BCT indicated in ERIC strategy
Develop disincentives	Provide financial disincentives for failure to implement or use the clinical innovations	2	14.1. Behaviour cost	Clear BCT indicated in ERIC strategy
			14.3. Remove reward	Clear BCT indicated in ERIC strategy
Use capitated payments	Pay providers or care systems a set amount per patient/consumer for delivering clinical care	1	10.2. Material reward (behaviour)	Clear BCT indicated in ERIC strategy
Make billing easier	Make it easier to bill for the clinical innovation	1	12.1. Restructuring the physical environment	Probable BCT indicated in ERIC strategy
Alter patient/consumer fees	Create fee structures where patients/consumers pay less for preferred treatments (the clinical innovation) and more for less-preferred treatments	1	14.8. Reward alternative behaviour	Probable BCT indicated in ERIC strategy
Fund and contract for the clinical innovation	Governments and other payers of services issue requests for proposals to deliver the innovation, use contracting processes to motivate providers to deliver the clinical innovation, and develop new funding formulas that make it more likely that providers will deliver the innovation	2	10.1. Material incentive (behaviour)	Probable BCT indicated in ERIC strategy
			1.8. Behavioural contract	Probable BCT indicated in ERIC strategy
Use other payment schemes	Introduce payment approaches (in a catch-all category)	2	12.5. Adding objects to the environment	Probable BCT indicated in ERIC strategy
			10.3. Material incentive (behaviour)	Probable BCT indicated in ERIC strategy
Access new funding	Access new or existing money to facilitate the implementation	2	12.5. Adding objects to the environment	Probable BCT indicated in ERIC strategy
			10.3. Material incentive (behaviour)	Probable BCT indicated in ERIC strategy
**Group 9: change infrastructure**			*4.Shaping knowledge* *6.Comparison of behaviour* *7. Associations* *9. Comparison of outcomes* *10.Reward and threat* *12.Antecedents* *14.Scheduled consequences*	
Create or change credentialing and/or licensure standards	Create an organization that certifies clinicians in the innovation or encourage an existing organization to do so. Change governmental professional certification or licensure requirements to include delivering the innovation. Work to alter continuing education requirements to shape professional practice toward the innovation)	4	12.1. Restructuring the physical environment	Probable BCT subsumed under ERIC strategy
			12.2. Restructuring the social environment	Probable BCT subsumed under ERIC strategy
			10.6. Non-specific incentive	Probable BCT subsumed under ERIC strategy
			4.1. Instruction on how to perform behaviour	Probable BCT subsumed under ERIC strategy
Change accreditation or membership requirements	Strive to alter accreditation standards so that they require or encourage use of the clinical innovation. Work to alter membership organization requirements so that those who want to affiliate with the organization are encouraged or required to use the clinical innovation	1	10.6. Non-specific incentive	Probable BCT indicated in ERIC strategy
Change service sites	Change the location of clinical service sites to increase access	1	12.1. Restructuring the physical environment	Clear BCT indicated in ERIC strategy
Change record systems	Change records systems to allow better assessment of implementation or clinical outcomes	1	12.1. Restructuring the physical environment	Probable BCT indicated in ERIC strategy
Change physical structure and equipment	Evaluate current configurations and adapt, as needed, the physical structure and/or equipment (e.g., changing the layout of a room, adding equipment) to best accommodate the targeted innovation	2	12.1. Restructuring the physical environment	Direct 1-1 overlap
			12.5. Adding objects to the environment	Clear BCT indicated in ERIC strategy
Change liability law	Participate in liability reform efforts that make clinicians more willing to deliver the clinical innovation	4	7.5. Remove adverse stimulus	Probable BCT indicated in ERIC strategy
			14.1 Behavioural cost	Probable BCT indicated in ERIC strategy
			14.2 Punishment	Probable BCT indicated in ERIC strategy
			10.1. Material incentive (behaviour)	Probable BCT indicated in ERIC strategy
Mandate change	Have leadership declare the priority of the innovation and their determination to have it implemented	2	9.1. Credible source	Probable BCT subsumed under ERIC strategy
			6.3. Information about others approval	Clear BCT subsumed under ERIC strategy
Start a dissemination organization	Identify or start a separate organization that is responsible for disseminating the clinical innovation. It could be a for-profit or non-profit organization	0	-	ERIC strategy not targeting behaviour change

For some strategies (*n*=18), one or more BCTs were clearly indicated in the description. For example, three BCTs were clearly indicated in the description for the ERIC strategy to *develop a formal implementation blueprint*. An implementation blueprint should *include the aim/purpose of the implementation and the scope of the change (e.g., what organizational units are affected), (3) timeframe and milestones, and (4) appropriate performance/progress measures* [7]. The BCT ‘action planning’ was clearly indicated as it refers to prompting detailed planning or performance of behaviour and must include at least one of context, frequency, duration, and intensity. ‘Goal-setting (behaviour)’, which refers to setting or agreeing a goal defined in terms of the behaviour to be achieved, was coded given references in the full description to the purpose of implementation, the scope of change, coordinating the blueprint with a fidelity monitoring tool and the types of intervention required at different organisational levels. ‘Goal setting (outcome)’, which refers to setting or agreeing a goal defined in terms of a positive outcome of wanted behaviour, was coded on account of reference to appropriate performance/progress measures.

More often, strategy descriptions indicated BCTs that were probable (*n*=31), that is logically indicated given the title, definition, and/or description of the strategy but not clearly or explicitly described. The designation of ‘probable’ reflected a lack of specification in the strategy description and the scope for strategies to operationalised with different degrees of change. The BCT ‘restructuring the physical environment’ was most often coded as probable as it was not clear to what extent strategies involved full-scale physical change. This applied to eight strategies: *develop and organise quality monitoring systems, develop and implement tools for quality monitoring, centralize technical assistance, use data warehousing techniques, facilitate relay of clinical data to providers, make billing easier, change record systems, create or change credentialing, and/or licensure standards*.

The descriptions of some strategies (*n*=15) indicated multiple types of overlap depending on the BCT being considered and the explicitness of the strategy description. This mix of clear and probable BCTs was evident across the nine ERIC strategy groups.

#### ERIC strategies with no BCTs indicated or not targeting behaviour change

Four ERIC strategy descriptions had no BCTs indicated explicitly or logically: *make training dynamic, assess for readiness and identify barriers and facilitators, promote adaptability, and develop educational materials*. Four ERIC strategies were categorised as not focusing on behaviour change for implementation: *conduct local needs assessment, develop an implementation glossary, work with educational institutions, and start a dissemination organisation*.

### Patterns of complementarity

Several themes were identified during the analysis that reflected different patterns of complementarity between ERIC strategies and BCTs.

#### Within a single strategy, there are different types of overlap with BCTs

Within a single strategy, there were multiple different types of overlap with different BCTs; as mentioned, 15 strategies were a mix of BCTs that were clearly indicated and others that were probable. Strategy descriptions contained explicit text which clearly indicated a BCT and non-specific text which suggested a BCT was probable and logical given the description. For example, the description of the strategy to *inform local opinion leaders* clearly indicated the BCT ‘identification of self as role model’ while ‘credible source’ was probable depending on the colleagues identified.

#### ‘Broad strategies’ indicate relatively few and broad BCTs but the list of probable BCTs is extensive

In the ERIC compilation, there were what we referred to as ‘broad strategies’ that is descriptions were short and/or not overly specific about what the strategy involved. Most strategies that were broadly defined indicated a single BCT in their description. The BCTs indicated were similarly broad in scope; typically ‘restructuring the physical environment’ or ‘restructuring the social environment’. Restructuring the physical environment was the only BCT indicated in four ERIC strategy descriptions (*change service sites, use data warehousing techniques, make billing easier, change record systems*). Restructuring the social environment was the only BCT indicated in six ERIC strategy descriptions (*build a coalition, create new clinical teams, revise professional roles, develop academic partnerships, recruit, designate and train for leadership, involve patients/consumers to enhance uptake and adherence*).

While broad strategies indicated few BCTs in their description, the list of probable BCTs was considered extensive and with limited information in the full description, and it was not possible to code further BCTs as clear or probable. For example, the strategy to *involve patients/consumers and family members* is described as engaging or including patients/consumers and families in the implementation effort, and the BCT restructuring the social environment was coded as probable. Other BCTs are probable depending on how this strategy is operationalised, but there was no information to inform further coding.

#### ERIC strategies serve as examples of BCTs operationalised for implementation

ERIC strategies, including some of those mentioned above, provide contextual examples of how BCTs could be operationalised for implementation. This pattern was evident for BCTs that we considered to be broadly defined, and these BCTs were among the most frequently coded; ‘restructuring the social environment’ (18 strategies: 7 clear indications and 10 probable indications), ‘credible source’ (12 strategies: 1 clear indication and 11 probable), restructuring the physical environment (9 strategies: 1 direct overlap, 1 clear, 8 probable), and ‘social support (practical)’ (8 strategies: 3 clear and 5 probable). The ERIC strategies provided examples as to how the same BCT could be operationalised in different ways to support implementation.

#### Presence of some BCTs is dependent on form of delivery

The presence/absence of some BCTs was dependent on the form of delivery indicated in the strategy description. For example, the BCT most frequently coded as probable was ‘credible source’ (for 12 strategies) as the presence/absence of this BCT is dependent on who delivers the information and whether they are credible to the target population. This BCT was coded as probable for all strategies except *providing ongoing consultation* which refers explicitly to the use of experts in the strategies to support implementation of the innovation.

Other ERIC strategy descriptions make suggestions about or imply the form of delivery. Strategies involving the BCT restructuring the social environment suggest operationalisation using either group delivery methods (e.g., *creating a learning collaborative*) or individual level delivery (e.g., *providing local technical assistance*). Other strategies suggest the setting where the strategy would be delivered (e.g., *visit other sites*) or delivery features relating to the provider (e.g., *identify and prepare local champions, provide clinical supervision*). The strategy *make training dynamic* refers to several elements of the form of delivery including delivery format and intensity which could implicate BCTs in the operationalisation of this strategy.

#### Some ERIC strategies are steps in the implementation process and target collective cognition at the system level

Eight ERIC strategies, categorised as having no BCTs indicated or not targeting behaviour change, demonstrated that some strategies are part of early phases of planning for implementation. For example, the strategies to *assess for readiness and identify barriers and enablers* and *promote adaptability* are overlapping/interrelated processes that could be used during pre-implementation to inform strategy or BCT selection. Their purpose is not to directly influence behaviour for implementation at the service-delivery level but rather to inform decision-makers and change minds at a system level.

Other strategies, which we categorised as not targeting behaviour change, are system-level processes focussed on information gathering or sharing (strategies: *work with educational institutions, start a dissemination organisation, develop an implementation glossary, conduct local needs assessment*). These strategies could also be part of the exploration or preparation stages of implementation but are not informing the selection of strategies for implementation or execution of the innovation itself.

Finally, there is a sequence inherent in some strategies without (and sometimes with) BCTs which would likely be coupled in practice. For example, *developing educational materials* alone does not indicate any BCT in its description but would most likely be coupled with *distributing educational materials*, a strategy with probable BCTs to shape knowledge and provide information about consequences (‘instruction on how to perform behaviour’ and ‘information about health consequences’).

## Discussion

The aim of this study was to examine the extent and nature of the overlap between the ERIC compilation of implementation strategies and the BCT Taxonomy. Overall, we identified complementarity rather than redundancy when integrating these two taxonomies. There was only one instance where the ERIC implementation strategy directly overlapped with a BCT; the ERIC strategy to *change physical structure and equipment* overlapped directly with the BCT ‘restructuring the physical environment’. Most strategy descriptions had BCTs that were clearly indicated, BCTs that were probable but not explicitly described, or indicated both types of overlap within a single strategy. This study can be considered a foundational step to move from general descriptions of implementation strategies to full and consistent description of actions.

### Enhancing levels of specificity

Some ERIC strategy descriptions contained BCTs that are clearly indicated but more that are probable, depending on how the strategy is operationalised. With limited information in the strategy description, it was not feasible to code an exhaustive list of all BCTs. A number of studies have highlighted that ERIC strategies vary in their level of specificity [40, 41]. It has been suggested that taxonomies such as the ERIC compilation do not possess the granularity and specificity the BCT Taxonomy contains [27]. Our results challenge this assumption as we identified mixed levels of granularity in both taxonomies. Similar to other studies [13, 27], in our analysis broadly defined BCTs such as ‘restructuring the social environment’, ‘restructuring the physical environment’, and ‘social support (practical)’ were among the most frequently coded in ERIC strategy descriptions. Some ERIC strategies were more granular, describing what was being restructured in the environment or, the nature of social support provided to implementers. As such, they provided contextual examples of how broad BCTs could be operationalised for implementation. The BCT taxonomy is intended to apply to any behaviour so these instances from the ERIC compilation may provide examples for inclusion/cross-referencing with future versions of the BCT taxonomy. The ERIC compilation could also serve as a basis for identifying additional BCTs that are not reflected in version one of the BCT taxonomy.

Overall, 44% of all possible BCTs were identified in ERIC strategy descriptions. For the remaining 56% of BCTs, it may not be logical to ever consider them in any implementation strategy (e.g., biofeedback) or they may apply to broad strategies that are currently under-specified. Considering how those underutilised BCTs could be incorporated may be an opportunity to enhance the description and novelty of some ERIC strategies.

### Moving towards mechanisms of action

Specifying the BCTs indicated in ERIC strategy descriptions provides a path by which we could begin to understand mechanisms of action. In implementation science, a mechanism of action is defined as a ‘process or event through which an implementation strategy operates to affect desired implementation outcomes’ [42] Using the Theory and Technique Tool developed to link BCTs to mechanisms of action [43–45], we can suggest mechanisms of action linked to BCTs that are indicated in ERIC strategies. For example, four BCTs were clearly indicated in the description of the ERIC strategy to *provide ongoing consultation*. There is evidence of a link between each of the four BCTs and at least one mechanism of action (MoA): ‘social support (unspecified)’/social influences (MoA), ‘credible source’/attitude toward behaviour and general attitudes and beliefs (MoAs), ‘feedback on behaviour’/motivation and feedback processes (MoAs), and ‘instruction on how to perform the behaviour’/knowledge, and skills and beliefs about capabilities (MoAs). Strategy-BCT-MoA linkages could provide the building blocks for testable causal models that can be refined over time. The MoA linkages mentioned here are based on those described in behaviour change interventions and coded using the Theoretical Domains Framework and additional MoA constructs from theories of behaviour change [43]. Mechanisms can operate at different levels including the organisational, community or macro policy level [42] and mechanisms at those levels may not be captured sufficiently by current tools which concentrate on the individual level. Efforts are underway to develop a research agenda to advance understanding of the mechanisms of implementation strategies [46].

### Form of delivery

While we did not formally code all elements of form of delivery, some ERIC strategy descriptions suggest or imply forms of delivery and as a result additional BCTs were coded. Delineating and describing the strategy and form of delivery is an important step when designing and reporting implementation interventions. Even with this distinction, the taxonomies examined in this study are not intended to deliver intervention content ready to use ‘off the shelf’. Other dimensions that also need to be clarified to fully operationalise an implementation strategy include the actors delivering the strategy, targets of the action, temporality, and dose (frequency and intensity) [1, 47].

The presence or absence of BCTs in a strategy description is not an indicator of strategy effectiveness. Furthermore, for some techniques, there is evidence of when and how they should be applied to ensure theoretical coherence and effectiveness, these characteristics should be persevered during operationalisation [47, 48]. In this analysis ‘credible source’ was one of the most common BCTs indicated in ERIC strategy descriptions. It was primarily coded as a probable BCT as it depends on the source of information and whether they are identified as credible by recipients of the information. According to Peters and colleagues there are a number of parameters of effectiveness for modelling, an implicit part of this BCT [48]: the recipient must attend to the communication, must remember it, and must have a sufficient skill to perform the desired behaviour; then, the recipients must identify with the model; the model must be positively reinforced for the desirable behaviour; and the model should be a coping model as opposed to a mastery model. When operationalising an ERIC strategy, it is not enough to consider whether the BCT is present or absent, we also need to consider how it is applied in practice to ensure it is functioning as intended. This is essential for understanding the mechanisms of action and inaction when assessing the effectiveness of implementation strategies.

### Deconstructing high-level and preparatory strategies

Some may consider that the BCT taxonomy and ERIC compilation focus, to varying degrees, on different levels of individual and organisational change, and reflect tensions between the traditions of behaviour-change-oriented health psychology and system-oriented organizational psychology/change management. We believe the separate application of the BCT taxonomy and ERIC compilation may constrain our thinking about how to best draw from both to describe implementation interventions. In this analysis, we demonstrate if and how the BCT taxonomy applies across all levels of ERIC strategies. Strategies can be deployed down the implementation chain to influence actions among frontline implementers and upwards to influence collective decision-making or action at an organisational and system level. Our analysis deconstructs some of the broad organisational strategies into behaviour change techniques to support implementation at these higher levels. It is argued better reporting of who needs to do what differently at a higher level would more comprehensively capture the multilevel changes involved in implementation [13]. In our analysis, nine ERIC strategies were categorised as having no BCTs indicated in their description or were not targeting behaviour change for the implementation of an innovation. The results align with recent efforts to organise implementation strategies into categories related to their timing [40] and target [49]. In terms of timing, strategies such as *assessing readiness* and *tailoring* could be used to prepare for the execution of the innovation or to inform the selection of other strategies. Vax et al. assigned these strategies, among others, to readiness stages of pre-contemplation, contemplation, and preparation [40]. In terms of target, we classified four strategies which did not target behaviour change but instead focussed on information gathering or information sharing at an organisational level (*work with educational institutions, start a dissemination organisation, develop an implementation glossary, conduct local needs assessment*). This grouping reflects aspects of the classification system proposed by Leeman and colleagues in which strategies are organised according to the strategy actors and action targets [49]. Within this system, dissemination strategies are one class of strategy which targets awareness, knowledge, attitudes, and intention to adopt an innovation. Thus, failure to identify BCTs in every strategy does not necessarily reflect a weakness of a strategy but may reflect a difference in the timing or target of a strategy.

Some of the ERIC strategies in this group may more closely reflect policy categories proposed in the behaviour change wheel approach to enable interventions [31]. Although not a formal part of this analysis, ERIC strategies such as working with educational institutions and starting a dissemination organisation appear to overlap with the policy category ‘environmental/social planning’, defined as designing and/or controlling the physical or social environment to enable interventions. Other policy categories in the BCW approach map to ERIC strategies in which specific BCTs were probable, for example, the ERIC strategy to use mass media and the policy category ‘communication/marketing’. Different aspects of the BCW approach (BCTs, intervention functions, policy categories) could be linked to ERIC strategies depending on the level of granularity required. This reflects the range of strategies at different levels of change outlined in the ERIC compilation.

### Strengths and limitations

We systematically coded ERIC strategy descriptions using the BCT Taxonomy and classified the level of overlap using a de novo typology. Other classification systems are used to design and specify implementation interventions which may also overlap with these taxonomies [8]. While guidance on coding intervention content using BCTs warns against making inferences or assumptions that a BCT is present unless there is evidence that it has been delivered [37], the purpose of this analysis was to examine the extent and nature of overlap between BCTs and strategies. Therefore, we considered it necessary to code probable BCTs to highlight the lack of specification in certain strategy descriptions. Additional BCTs beyond those coded in this analysis are possible as part of any strategy, depending on how it is operationalised in a given context.

One researcher coded all the ERIC strategy descriptions using the BCT taxonomy. To minimise the influence of researcher assumptions or familiarity with certain strategies, three analysts reviewed and rated their agreement with the coding. They also provided suggestions of BCTs that should be removed or added.

We recognize that depending on how any given ERIC strategy is operationalized, there could be many other potential BCTs possible. Instead of making assumptions, we opted for a conservative approach in our analysis and coded the strategy definition and detailed description in the ERIC compilation *only*. It is likely these descriptions are a starting point for how a strategy could be operationalised, rather than an exhaustive or prescriptive description. Primary studies of implementation interventions are another source of information on how strategies are operationalised by researchers and implementers. BCT-coding intervention descriptions in future systematic reviews could be another step in the process of specifying ERIC strategies. Researchers have begun to synthesize the content of commonly used strategies [22, 27]. BCT coding is dependent on the richness of the strategy description [13] and the specificity of the BCT definitions. As mentioned previously, broadly defined BCTs were the most frequently coded in ERIC strategy descriptions which could reflect the ease with which their definitions are identifiable in the text.

The results of this study have a number of practical implications. First, the study distinguishes between ERIC strategies based on the extent to which they are similar to or indicate BCTs and thus can be readily operationalised. It places strategies on a continuum from clearly specified to those requiring more work before application. This could be potentially useful to researchers and practitioners trying to design, replicate, scale, and spread implementation interventions. These frameworks could be integrated iteratively in several ways. For example, designers could begin with the ERIC compilation to name strategies in a language that is accessible to stakeholders and specify the activities within those strategies using the BCT taxonomy, using the coding in this analysis as a starting point. Alternatively, designers using the behaviour change wheel approach to intervention design could refer to the ERIC compilation to see how certain intervention functions or BCTs could be operationalised in an implementation context. Second, the results could also inform fidelity assessment by suggesting observable BCTs that could be monitored as strategies are deployed [50, 51]. Finally, each taxonomy can make a unique contribution that can be leveraged to enhance the reporting, replication, and synthesis of strategies. The ERIC compilation provides an accessible language for community partners and practitioners designing implementation strategies. Integrating the BCT taxonomy encourages consideration of the actions and targets of these strategies.

## Conclusion

The myriad of theories, models, and frameworks is an accepted part of implementation science. However, there is increasing recognition of the opportunity and needs to combine these tools [52]. This study highlights the complementarity rather than redundancy that can come from combining the ERIC compilation and BCT taxonomy. Each taxonomy can make a unique contribution to enhance the reporting, replication, and synthesis of strategies. More efforts to integrate these taxonomies will aid strategy developers and build links between existing silos in implementation science.

## Supplementary Information

**Additional file 1.**

## Data Availability

Not applicable

## Abbreviations

AIMD: Aims, Ingredients, Mechanism, Delivery
BCT: Behaviour change technique
ERIC: Expert Recommendations for Implementing Change
StaRI: Standards for Reporting Implementation Studies
TIDieR: Template for Intervention Description and Replication
